# Translabyrinthine Petrous Apex Cholesteatoma Surgery with Hearing Preservation

**DOI:** 10.1155/2021/5541703

**Published:** 2021-06-12

**Authors:** Holger Sudhoff, Randolf Klingebiel, Lars-Uwe Scholtz, Ingo Todt

**Affiliations:** ^1^Department of Otorhinolaryngology, Head and Neck Surgery, Medical Faculty OWL, Bielefeld University, Campus Klinikum Bielefeld, Bielefeld, Germany; ^2^Department of Diagnostic and Interventional Neuroradiology, Protestant Hospital Bethel, Bielefeld, Germany

## Abstract

**Objective:**

To introduce a novel surgical approach to petrous apex lesion (PA) with superior semicircular canal plugging for hearing preservation. *Patient*. A 63-year-old patient presented with a recurrent cholesteatoma of the left petrous apex. The patient had a long-term history of cholesteatoma and MRI with diffusion-weighted imaging (DWI) detected a suspicious lesion in the left petrous apex on follow-up. *Intervention*. The cholesteatoma could be completely removed from the petrous apex with partial superior semicircular canal plugging and removal with hearing preservation. *Outcomes*. Cholesteatomas of the temporal bone are managed by surgery with complete excision of the lesion.

**Results:**

The translabyrinthine approach, generally useful in nonhearing ears, could be utilized with the additional technique of superior semicircular canal plugging to preserve hearing in this patient.

**Conclusions:**

This case highlights the possibility of a hearing preservation strategy for PA cholesteatomas using a translabyrithine approach.

## 1. Introduction

The petrous apex (PA) is embedded in the medial portion of the temporal bone, bordered by several vital structures [1]. A variety of different petrous apex pathologies such as cholesterol granulomas, cholesteatomas, meningiomas, and schwannomas have been described [2]. Cholesterol granulomas are significantly more common than cholesteatomas of the petrous apex, comprising the most frequent destructive lesion of the petrous apex. The surgical options for petrous apex cholesteatoma use open, endoscopic, or combined techniques. The specific approach is determined by the pathology and its anatomical localization and extension related to vital structures. Individual anatomical variations in cranial morphology may interfere with different surgical approaches. Removal of the lesion can be achieved through the transcanal infracochlear, transmastoid infralabyrinthine, middle fossa, translabyrinthine, transotic approach, or occasionally by a transsphenoidal approach [3–5]. However, there is a need for a novel surgical strategy to petrous apex lesion (PA) with hearing preservation as a regular approach usually linked to a loss of hearing and decrease of quality of life [6, 7]. The recently introduced superior semicircular canal plugging for hearing preservation seems as a rational for this translabyrinthine approach [6].

**Figure 1:**
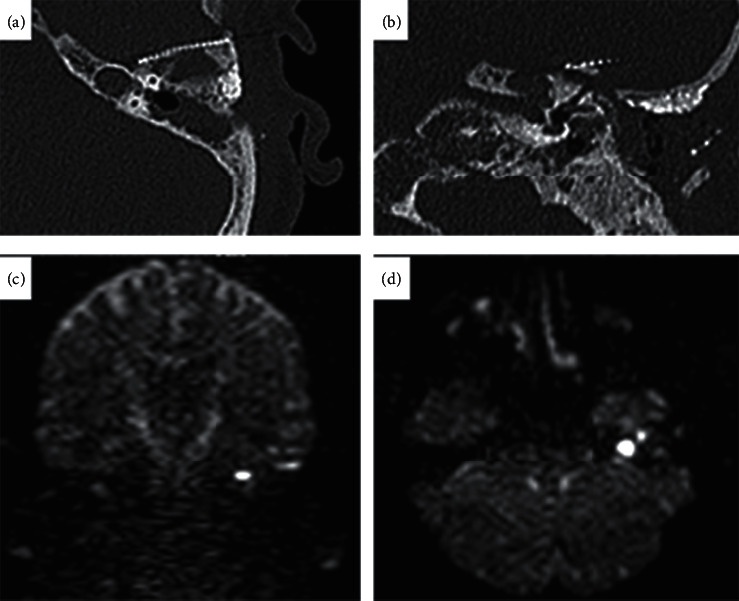
(a, b) Preoperative axial CT showing (asterixs) the petrous apex with bone erosion and a titanium plate covering the partially covering anterior limitation of the temporal bone. (c, d) Non-EPI DWI with coronal and axial planes. Note the diffusion restriction in the petrous apex of the left temporal bone.

PA pathologies are usually found incidentally on imaging for unrelated symptoms. If present, they are heterogeneous. The most common complaint is hearing loss, but aural fullness and vertigo secondary to compression of the 8th cranial nerve are also common. These symptoms are present in the majority of patients with petrous apex pathology. Additional symptoms include headaches due to the involvement of the trigeminal nerve, diplopia from compression of the sixth cranial nerve, and seventh-nerve-related facial spasm or weakness. Extended cholesteatoma may also result in Gradenigo's syndrome including purulent otorrhea, abducens palsy, and otalgia [6]. Evaluation of PA lesions involves a comprehensive evaluation of cranial nerve function and audiometry. Computed tomography (CT) is routinely used to determine the optimal surgical approach if surgical intervention is necessary (Figure 1). Magnetic resonance imaging (MRI) can narrow the differential diagnosis. In our case, a 63-year-old patient presented with a cholesteatoma of the left petrous apex six years after extended middle ear surgery on follow-up images. MRI with diffusion-weighted imaging (DWI) detected a suspicious lesion in the left petrous apex (Figure 1).

## 2. Case

**Figure 2:**
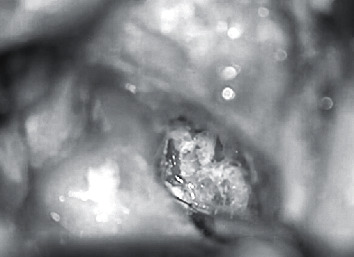
Intraoperative image showing the petrous apex with the cholesteatoma matrix prior to removal. The superior semicircular canal has been plugged, sealed, and partly amputated.

**Figure 3:**
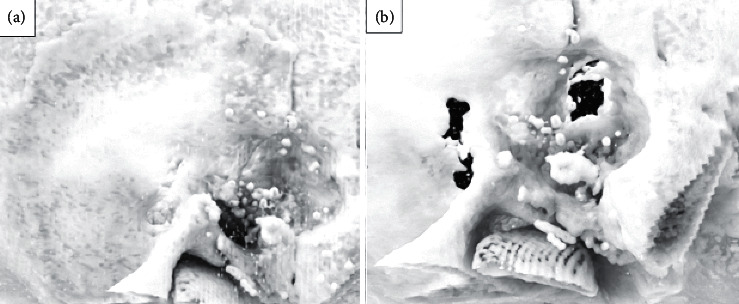
3D reconstruction of CTs. (a) The preoperative scan reconstruction. (b) The postoperative scan exhibiting the defect of the petrous apex after the partial translabyrinthine approach.

**Figure 4:**
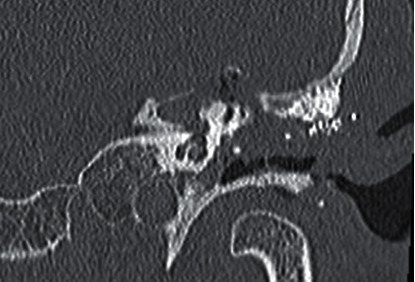
Coronal CT of the amputated superior semicircular canal (asterix) and the postoperative defect of the left petrous apex.

**Figure 5:**
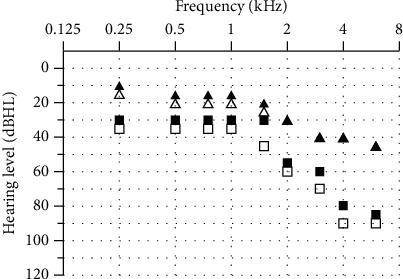
Left ear, pre- (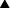 air conduction and 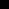 bone conduction) and postoperative (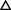 air conduction and 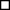 bone conduction) pure-tone audiogram. Hearing thresholds remained on the operated left ear one year after surgery.

The basic goal of cholesteatoma surgery is the complete removal of the squamous epithelium to minimize the risk of recurrence. Cholesteatoma is a life-threatening disease due to its intracranial complications [7]. Due to an expected inner ear trauma during surgery, 1 g of prednisolone was administered in addition to the antibiotic treatment preoperatively. Facial nerve monitoring was constantly applied. A retroauricular incision was used in this patient. It allowed extensive exposure of the mastoid. The mastoid was partially obliterated with hydroxyapapatite granules but did not reveal the cholesteatoma matrix. A titanium net had been implanted to stabilize the middle and posterior cranial fossae in a revision surgery six years earlier (Figure 1). An extended mastoidectomy was performed. The sinus, media, and posterior fossa were identified as well as the temporarily removed total ossicular chain prosthesis. The semicircular canals were detected and exposed. The common crus of the posterior and superior canals was identified. Finally, the bone between the superior canal and tegmen was carefully drilled away layer by layer with a small diamond burr until the blue lines of the superior canal were visible. The dura and the titanium plate were slightly elevated to expose the superior canal. The endosteal islands of the superior semicircular were elevated and the membranous labyrinth visualized. The plug was a mix of dry cortex bone dust and fibrinogen sealant. This was carefully packed into both fenestrations of the superior semicircular canal to occlude the superior, lateral, and medial portion. This created a partition from the rest of the inner ear. Temporalis fascia was placed over the occlusions and covered with a fibrinogen sealant to protect against perilymph leakage. After the occlusion and removal of the superior aspect of the semicircular canal, sufficient access was granted to the petrous apex. Hopkin's wide-angle endoscope was used to control the extension and removal of the cholesteatoma of the petrous apex that could not be assessed with the microscope (Figures 2 and 3). All cholesteatoma matrixes were eliminated from the petrous apex (Figure 4). The preoperative hearing threshold could be preserved by superior semicircular canal plugging (Figure 5). Histopathology confirmed a cholesteatoma of the petrous apex. Postoperatively, the patient was stable with preserved hearing thresholds on the left ear and declining dizziness with a loss of vestibular function on one-year follow-up.

## 3. Discussion

Semicircular canal plugging was originally introduced to treat patients with intractable benign paroxysmal positional vertigo and was subsequently applied to other hydropic ear diseases such as Meniere's disease [8, 9]. Semicircular canal plugging can reduce vertiginous symptoms in those patients representing an effective therapy for this disorder. Therefore, it seems possible to utilize the additional technique of superior semicircular canal plugging, to preserve hearing in partial translabyrinthine surgery. Previously, a modified traditional translabyrinthine approach for the removal of an intracanalicular acoustic schwannoma by sealing the vestibule with bone wax allowed the hearing function to be preserved in one patient [10, 11]. The described translabyrithine approach for hearing preservation is currently limited to selected cases with constrained pathologies, such as petrous apex cholesteatomas [6, 12, 13]. Due to the very limited experience with a partial removal of the superior semicircular canal, patients have to be consented about deafness, significant hearing loss, and vertigo. The use of Hopkin's wide-angle endoscope was necessary in the petrous apex that could not be completely assessed with the microscope. Computed tomography of the temporal bone is helpful in revealing a possible residual cholesteatoma and to assess the extension, exact location, and possible complications. Nonecho planar diffusion-weighted magnetic resonance imaging (non-EPI DW MRI) is a powerful tool to detect of localization and extension of cholesteatoma [14]. Due to a possible inner ear trauma during surgery, steroids and antibiotic prophylaxis should be administered. Facial nerve monitoring is recommendable during the entire procedure.

The translabyrinthine approach provides the straightest route to the petrous apex [1, 6, 13]. However, it has previously been considered unsuited for hearing preservation. Therefore, it was limited to cases where hearing and vestibular function were absent. Our case highlights the possibility to grant access to the petrous apex region and to preserve hearing in partial labyrinthectomy. The additional application of superior semicircular canal plugging, sealing, and partial removal provides the possibility to preserve hearing in selected patients [15]. The case underlines this surgical option in selected skull base pathologies requiring limited access to the petrous apex.

## 4. Conclusions

A limited number of studies appear to demonstrate the possibility of hearing preservation in patients after translabyrinthine cholesteatoma removal. Therefore, this case report further highlights the potential mechanisms for hearing preservation after labyrinthectomy. However, further studies are required to implement this approach to preserve hearing for extended cholesteatomas of the petrous apex region.
